# Supplementary material for: Cancer Cell Secreted Legumain Promotes Gastric Cancer Resistance to Anti-PD-1 Immunotherapy by Enhancing Macrophage M2 Polarization
Source: Pharmaceuticals (Basel). 2024 Jul 16;17(7):951. doi: 10.3390/ph17070951 (PMC11279811; doi:10.3390/ph17070951)
Supplement: Supplementary file 1 [file pharmaceuticals-17-00951-s001.zip › pharmaceuticals-3053385-supplementary.pdf]

Supplementary Figure S1

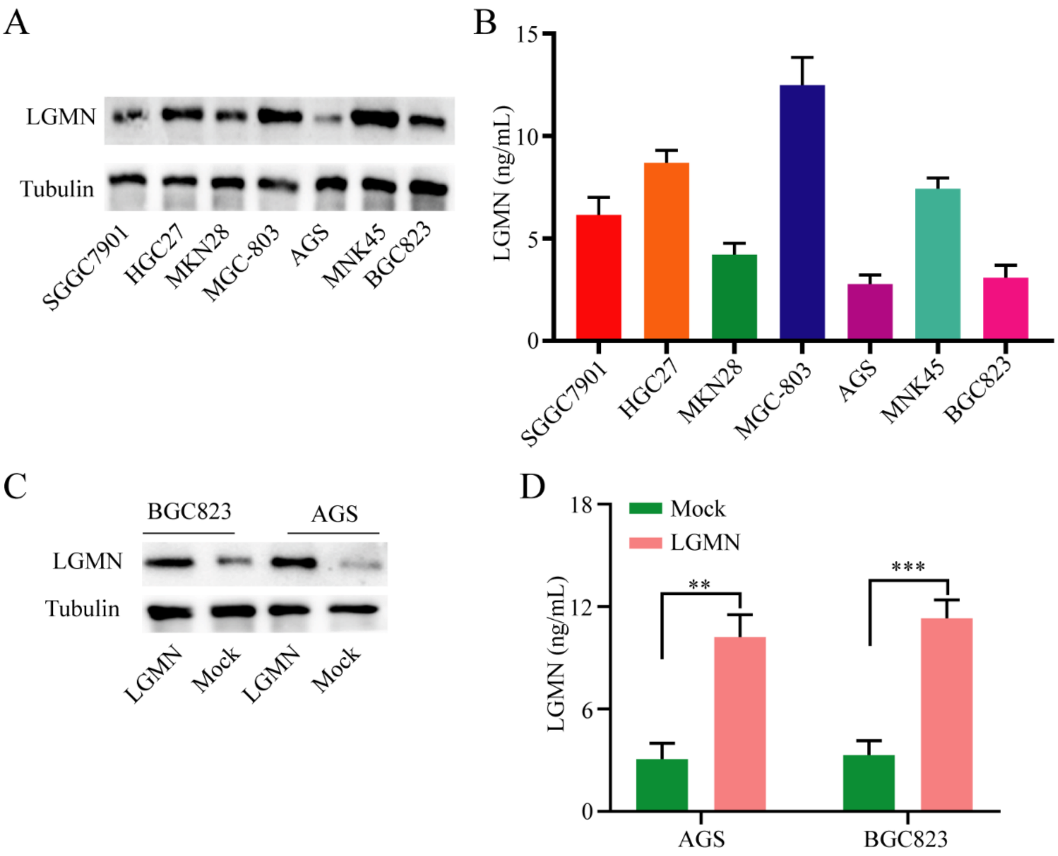

Supplementary Figure S1. Establishment of LGMN overexpressing gastric cancer cell lines. A. Western blot analysis of LGMN expression levels in 7 gastric cancer cell lines. B. ELISA detection of LGMN expression levels in the supernatant of 7 gastric cancer cell line cultures. C. Lentiviral vectors were used to upregulate LGMN expression in AGS and BGC823 cells, establishing LGMN overexpressing cell lines (AGS-L and BGC823-L). D. ELISA detection of LGMN expression levels in the supernatant of AGS-L and BGC823-L cells as well as control cells (Mock). ***P<0.001; **P<0.01.
